# The Hypothesis of the Human iNKT/Innate CD8(+) T-Cell Axis Applied to Cancer: Evidence for a Deficiency in Chronic Myeloid Leukemia

**DOI:** 10.3389/fimmu.2016.00688

**Published:** 2017-01-16

**Authors:** Florence Jacomet, Emilie Cayssials, Alice Barbarin, Deborah Desmier, Sara Basbous, Lucie Lefèvre, Anaïs Levescot, Aurélie Robin, Nathalie Piccirilli, Christine Giraud, François Guilhot, Lydia Roy, André Herbelin, Jean-Marc Gombert

**Affiliations:** ^1^INSERM 1082, Poitiers, France; ^2^Service d’Immunologie et Inflammation, Poitiers, France; ^3^CHU de Poitiers, Poitiers, France; ^4^Université de Poitiers, Poitiers, France; ^5^Service d’Hématologie et d’Oncologie Biologique, Poitiers, France; ^6^Etablissement Français du Sang Centre-Atlantique, Site de Poitiers, Poitiers, France; ^7^INSERM CIC-1402, Poitiers, France; ^8^Service d’Hématologie Clinique, Hôpital Henri Mondor, Créteil, France; ^9^Université Paris-Est, Créteil, France

**Keywords:** innate CD8(+) T cells, NK-like CD8(+) T cells, iNKT cells, chronic myeloid leukemia, tyrosine kinase inhibitor

## Abstract

We recently identified a new human subset of NK-like [KIR/NKG2A(+)] CD8(+) T cells with a marked/memory phenotype, high Eomesodermin expression, potent antigen-independent cytotoxic activity, and the capacity to generate IFN-γ rapidly after exposure to pro-inflammatory cytokines. These features support the hypothesis that this new member of the innate T cell family in humans, hereafter referred to as innate CD8(+) T cells, has a role in cancer immune surveillance analogous to invariant natural killer T (iNKT) cells. Here, we report the first quantitative and functional analysis of innate CD8(+) T cells in a physiopathological context in humans, namely chronic myeloid leukemia (CML), a well-characterized myeloproliferative disorder. We have chosen CML based on our previous report that IL-4 production by iNKT cells was deficient in CML patients at diagnosis and considering the recent evidence in mice that IL-4 promotes the generation/differentiation of innate CD8(+) T cells. We found that the pool of innate CD8(+) T cells was severely reduced in the blood of CML patients at diagnosis. Moreover, like iNKT and NK cells, innate CD8(+) T cells were functionally impaired, as attested by their loss of antigen-independent cytotoxic activity and IFN-γ production in response to innate-like stimulation with IL-12 + IL-18. Remarkably, as previously reported for IL-4 production by iNKT cells, both quantitative and functional deficiencies of innate CD8(+) T cells were at least partially corrected in patients having achieved complete cytogenetic remission following tyrosine kinase inhibitor therapy. Finally, direct correlation between the functional potential of innate CD8(+) T and iNKT cells was found when considering all healthy donors and CML patients in diagnosis and remission, in accordance with the iNKT cell-dependent generation of innate CD8(+) T cells reported in mice. All in all, our data demonstrate that CML is associated with deficiencies of innate CD8(+) T cells that are restored upon remission, thereby suggesting their possible contribution to disease control. More generally, our study strongly supports the existence of an innate iNKT/innate CD8(+) T-cell axis in humans and reveals its potential contribution to the restoration of tumor immune surveillance.

## Introduction

A hallmark of the antigen-specific T lymphocytes of the adaptive immune system is their capacity to “remember” foreign pathogens long after they are first encountered. Indeed, as far as the CD8 T cell pool is concerned, the main features that provide antigen-specific memory CD8(+) T cells with a protective advantage over naive CD8+ T cells are that memory CD8+ T cells persist for extended periods of time, are present in larger numbers, and respond more rapidly to foreign antigen than naive CD8(+) T cells do. However, studies in mice have provided clear evidence that some naive CD8(+) T cells can acquire the characteristics and functions of memory CD8(+) T cells in the absence of foreign antigen encounters [reviewed in Ref. (1, 2)]. Such cells, hence called innate/memory CD8(+) T cells, may develop in response to alterations in the environment or the presence of high levels of the cytokine IL-4, and have also been identified in unmanipulated animals. A hallmark of innate/memory CD8(+) T cells is their marked NK-like/memory phenotype associated with pronounced expression of the transcription factor eomesodermin (Eomes) (3, 4). Even though the actual physiological significance of innate/memory CD8(+) T cells remains to be established, these cells have enhanced response potential, including the ability to efficiently combat pathogens (5, 6). Moreover, because these cells can rapidly produce large amounts of IFN-γ in response to innate-like stimulation by IL-12 + IL-18 (7, 8), they might contribute to the inflammation milieu during an early stage of immune response.

The existence of innate/memory CD8(+) T cells in mice raises the question whether an equivalent to these innate T cells exists in humans. Earlier studies identified a subset of CD8(+) T cells in human peripheral blood that express NK-cell receptors such as killer cell Ig-like receptors (KIRs) (9–11). We further demonstrated that the majority of KIR/NKG2A(+) CD8(+) T cells have a memory phenotype and share functional and phenotypic features (12), see commentary (13): with innate/memory T CD8(+) cells in mice (3, 4, 7, 8). Indeed, they express high levels of Eomes and display innate functions, such as cytolytic capacity and rapid TCR-independent IFN-γ production in response to the innate cytokines IL-12 and IL-18. Regardless of their origin, KIR/NKG2A(+) Eomes(+) CD8(+) T cells harboring memory phenotype and innate-like functions have also been identified in human cord blood, suggesting that their development did not depend on cognate antigens. Additionally, the presence of these cells correlated well with expression of promyelocytic leukemia zinc finger (PLZF) among NKT cells in cord blood, a finding that is consistent with the hypothesis of a contribution of IL-4-producing, PLZF-expressing cells to their generation, as reported for their equivalents in mice (14, 15). These features validate the existence of a new member of the innate T cell family in humans, which we have termed innate CD8(+) T cells. However, to date, even though evidence accounts for their potential physiological relevance, whether innate CD8(+) T cells can mediate potent immunity in humans remains to be investigated.

Chronic myeloid leukemia (CML) is a well-characterized myeloproliferative disorder, which results from dysregulated tyrosine kinase (TK) activity of the fusion oncoprotein BCR-ABL (16). Imatinib mesylate (IM), a competitive inhibitor of the BCR-ABL TK activity, is currently used as a first-line therapy for newly diagnosed patients in the chronic phase (CML-CP) (17). Overall, TK inhibitor (TKI) therapies have led to deep molecular responses in CML, dramatically improved life expectancy, and more recently, successful treatment-free survival has become a new goal (18). However, a significant proportion of patients will not fulfill the conditions allowing for treatment discontinuation while more than 40% of those eligible for treatment discontinuation relapse during the first 6 months after therapy cessation. Additional therapeutic strategies aiming at a potentiation and/or restoration of immune antitumor functions, therefore, remain a main avenue of research, which may be helpful in long-term control of CML.

A sizable number of clinical and experimental data underscore the dysregulation of innate immune components in CML. These regulatory elements comprise dendritic cells (19), NK cells (20, 21), and invariant natural killer T (iNKT) cells (22), an innate T cell subset co-expressing activated/memory and NK markers, which is well-recognized for its antitumor activity (23–26). In accordance with this notion, we recently reported CML immune subversion of iNKT-cell activities in CML patients at diagnosis (22, 27), including reduced or suppressed expression of perforin, CD95L, and (PLZF), a transcription factor required for maintenance of iNKT cell functions (28, 29). Remarkably, these functional deficiencies were shown to have been partially repaired in patients having achieved complete cytogenetic remission (CCyR) following TKI therapy (22).

In mice, IL-4-producing/PLZF-expressing cells, including iNKT cell, have been demonstrated to regulate the generation of innate/memory CD8(+) T cells (14, 15). Considering the functional deficiencies of iNKT cells in CML-CP patients at diagnosis and the fact that IL-4 production by iNKT cells was partially restored in patients having achieved CCyR after TKI therapy (22), we surmised that innate CD8(+) T cells might also be altered during CML and be restored in CML patients with CCyR. We further attempted to correlate the presence of innate CD8(+) T cells with that of iNKT cells to estimate the possible implication in humans of the peripheral iNKT cell pool in the development of its CD8(+) T-cell counterpart.

## Materials and Methods

### Peripheral Blood Mononuclear Cells (PBMCs)

Venous blood from CML-CP patients at diagnosis or having achieved a major molecular response and currently treated with IM (CML-IM) was collected on heparin (Oncology-Hematology Department, Poitiers, France). All patients gave informed consent in accordance with the Declaration of Helsinki for participation in the study, which was approved by the scientific committee of the INSERM CIC-1402 (Poitiers, France). Healthy donors (HDs) were volunteers from the Pôle Biologie Santé (Poitiers, France). PBMCs were isolated from blood samples by density gradient centrifugation (Histopaque^®^-1077, Sigma-Aldrich), resuspended in 90% fetal calf serum with 10% DMSO, and placed in a controlled rate freezer for cryopreservation at −80°C until use.

### Cell Culture and Functional Assays

All cell cultures (1 × 10^6^ cells/mL) were performed in RPMI 1640 medium supplemented with 10% heat-inactivated FCS and antibiotics. For IL-12 + IL-18 stimulation, PBMCs from HD or CML patients were seeded at 1 × 10^6^ cells/mL into 24-well plates and incubated for 48 h with 20 ng/mL of each cytokine (R&D Systems). Golgistop (BD Biosciences) was added for the last 5 h of culture. CD107a degranulation assays were performed as previously described (12). Briefly, PBMCs were seeded into 96-well round (*U*) bottom culture plates, preincubated for 48 h with IL-15 (20 ng/mL, R&D Systems) prior to CD16 triggering, and Golgistop (BD Biosciences) was added in the last 4 h of culture. For IL-4 stimulation, PBMCs from HDs were seeded at 0.5 × 10^6^ cells/mL into 24-well plates and incubated for 7 days with 20 ng/mL of recombinant human IL-4 (R&D Systems).

### Flow Cytometry

Phenotypic analysis of PBMCs was performed by flow cytometry either *ex vivo* or after culture. Expression of different markers was assessed by staining with appropriate combinations of the following antibodies (mAbs): anti-CD3 BV421 (clone: UCHT1, BioLegend), anti-CD8 PE-Cy7 (clone: RPA-T8, Biolegend), anti-IFN-γ FITC (clone: B27, BioLegend), anti-perforin FITC (clone: δG9, BD Biosciences), anti-TCR Vα24-Jα18 APC (clone: 6B11, Biolegend), anti-CD107a FITC (clone H4A3, BD Biosciences), anti-Eomes eFluor^®^ 660 (clone: WD1928, eBiosciences), and anti-PLZF PE (clone: Mags.21F7, eBioscience). Pan-KIR/NKG2A referred to staining with the mix of the three following antibodies from Miltenyi Biotech: anti-KIR2D PE (clone: NKVFS1), anti-KIR3DL1/KIR3DL2 (CD158e/k) PE (clone: 5.133), and anti-NKG2A (CD159a) PE (clone: REA110). Dead cells were excluded by using the Live/Dead^®^ Fixable Near-IR Dead Cell Stain kit (Life Technologies). For nuclear Eomes or PLZF staining and intracytoplasmic IFN-γ or perforin staining, cells were permeabilized with an anti-human Foxp3 staining kit (eBioscience) and a Cytofix/Cytoperm kit (BD Biosciences), respectively. Cells were analyzed by eight-color flow cytometry (FACSVerse™ cytometer and FACSuite™ software, BD Biosciences) and were analyzed using FlowJo v10 (TreeStar, Inc.). Innate CD8(+) T cells are defined as CD3(+) CD8(+) Eomes(+) KIR/NKG2A(+) and iNKT cells as CD3(+) TCRVα24-Jα18(+)-expressing cells after gating on live PBMCs.

### Statistical Analysis

Statistical analyses were performed using GraphPad Prism version 6.0 (GraphPad Software). The statistical significance of differences in mean values was analyzed by the Mann–Whitney or Wilcoxon non-parametric test. The correlation Spearman test was used to test the association between the ranked variables Eomes and PLZF. Results were considered to be statistically significant when *p* < 0.05.

## Results

### Quantitative and Functional Deficiencies of Innate CD8(+) T Cells from CML-CP Patients

**Figure 1:**
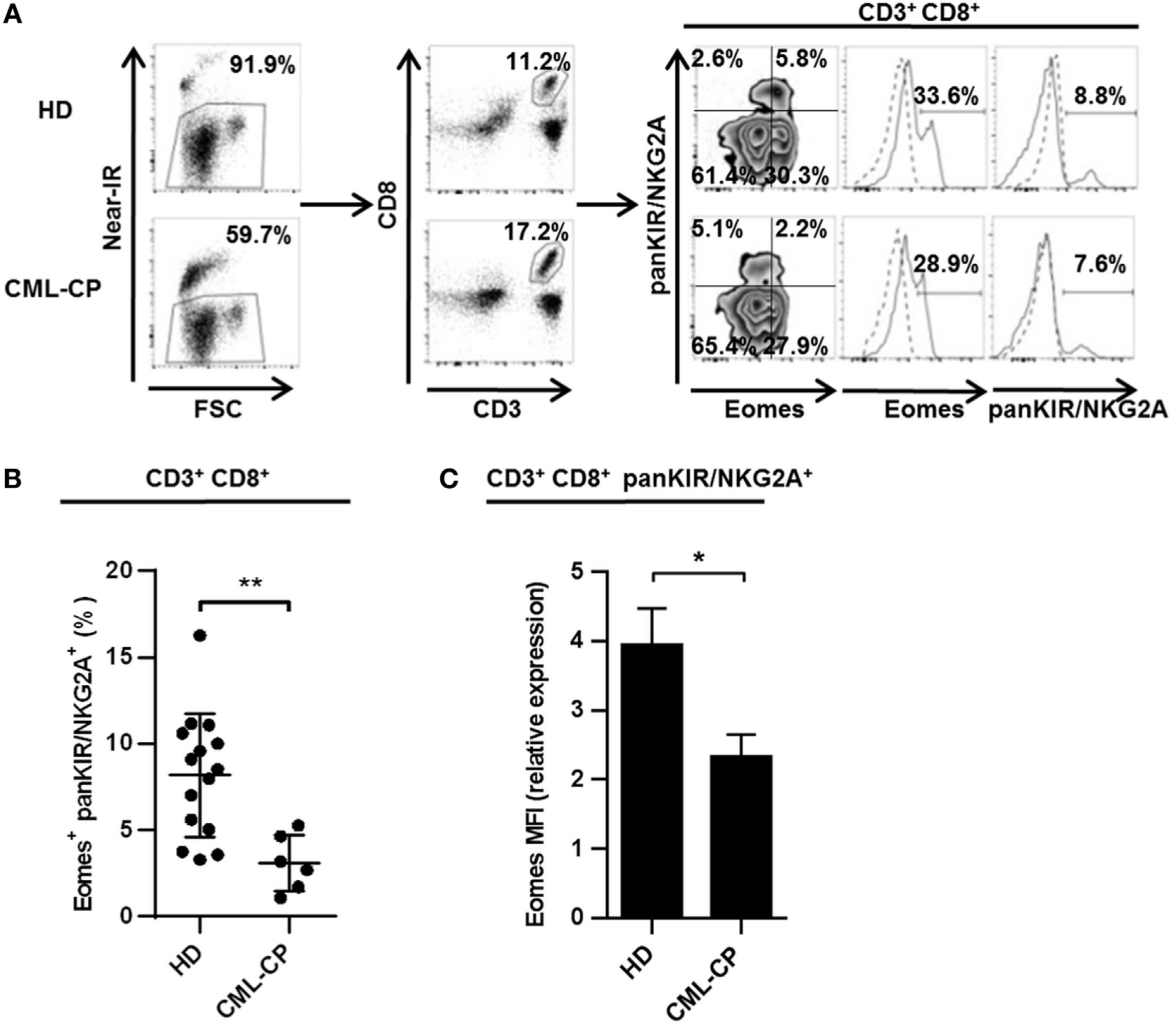
**Chronic myeloid leukemia (CML)-CP is associated with acquired quantitative defects of innate CD8(+) T cells**. **(A)** Gating strategy. Eomes and killer cell Ig-like receptor (KIR)/NKG2A expression among peripheral blood mononuclear cells was analyzed by flow cytometry after gating on live cells (Near-IR) and then on CD8(+) CD3(+) populations. Quantile contour plots show one representative sample for each group of healthy donor (HD) or CML-CP patients. Solid and dotted lines on histograms represent Eomes or pan-KIR/NKG2A expression or isotype control, respectively. **(B)** Decreased innate CD8(+) T cell counts in CML-CP patients. Frequency (mean ± SEM) of KIR/NKG2A(+) Eomes(+) cells among total CD8(+) CD3(+) cells in HD and CML-CP patients. Each dot represents one HD or CML-CP patient. **(C)** Decreased Eomes expression in CD3(+) CD8(+) KIR/NKG2A(+) T cells in CML-CP patients. Mean fluorescence intensity (MFI) of Eomes expression (mean ± SEM) in KIR/NKG2A(+) CD8(+) T cells from HD and CML-CP patients were analyzed after gating on KIR/NKG2A(+) CD8(+) CD3(+) cells. MFI of Eomes expression was normalized on MFI of Eomes from total KIR/NKG2A(−) CD3(−) cells. Statistical significance was determined by the two-tailed Mann–Whitney non-parametric test. *p < 0.05; **p < 0.01.

CD8(+) T cells co-expressing Eomes and KIR/NKG2A represent a new, functionally distinct “innate” subset in humans, with potential antitumor activities (12, 13). Based on our evidence for CML immune subversion of iNKT-cell activities (22, 27), we first investigated possible dysfunctions of this new innate CD8 T subset (for gating strategy, see Figure 1A) in CML patients at diagnosis (CML-CP). As depicted in Figure 1B, the frequency of KIR/NKG2A(+) Eomes(+) CD8(+) T cells was more than 2.5-fold lower in CML-CP patients (3.1% ± 0.7; *n* = 6) than in HDs (8.2% ± 0.9; *n* = 15).

Note that both the proportion of cells expressing Eomes among the KIR/NKG2A(+) CD8(+) T cell subset (Figure S1 in Supplementary Material) and the expression levels (Figure 1C) were significantly reduced in CML-CP patients [frequency: 26.3% ± 3.2 (*n* = 6) and mean fluorescence intensity (MFI): 2.17 ± 0.25 (*n* = 6), respectively] as compared to HD [frequency: 46.5% ± 4.6 (*n* = 14) and MFI: 3.95 ± 0.52 (*n* = 15), respectively]. Taken together, these data are consistent with impaired differentiation of the innate CD8 T cell subset in CML-CP patients.

**Figure 2:**
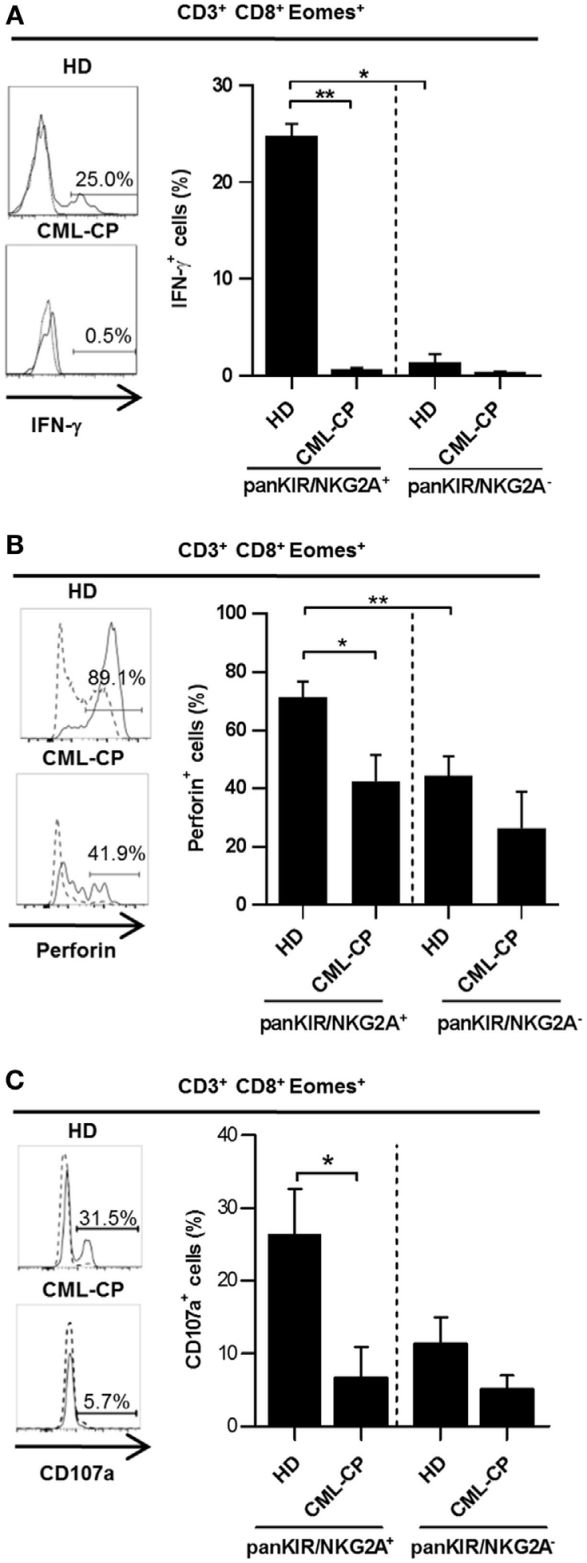
**Chronic myeloid leukemia (CML)-CP is associated with acquired functional defects of innate CD 8(+) T cells**. Peripheral blood mononuclear cells were cultured and stimulated for 48 h with IL-12 + IL-18 **(A,B)** or IL-15 prior to CD16 triggering **(C)** (see Materials and Methods). IFN-γ **(A)**, perforin **(B)** expression, or CD107a-expressing cells **(C)** (mean ± SEM) were analyzed after gating on killer cell Ig-like receptor (KIR)/NKG2A(+) Eomes(+) CD8(+) CD3(+) cells and KIR/NKG2A(−) Eomes(+) CD8(+) CD3(+) cells in healthy donor (HD) and CML-PC patients. Representative histograms of IFN-γ and perforin expression or CD107a-expressing cells are shown for KIR/NKG2A(+) Eomes(+) CD8(+) CD3(+) cells (filled line) or KIR/NKG2A(−) Eomes(+) CD8(+) CD3(+) cells (dotted line) for each group of HD and CML-CP patients. Without stimulation, frequency of IFN-γ expressing cells was lower than 0.1%. Full cohort data are shown. **(A–C)** Statistical significance to assess differences between populations in HD group or between HD and CML-CP was determined by the two-tailed Wilcoxon or Mann–Whitney non-parametric test, respectively. *p < 0.05; **p < 0.01.

We have previously reported (12) and have confirmed in our present HD cohort (Figure 2A) that the rapid production of IFN-γ in response to innate-like stimulation by IL-12 + IL-18 constitutes a unique hallmark of innate CD8(+) T cells. Indeed, this functional reactivity to IL-12 + IL-18 was found in the innate KIR/NKG2A(+) Eomes(+) fraction (24.8% ± 1.2; *n* = 6) and not in the conventional/memory KIR/NKG2A(−) Eomes(+) pools of CD8(+) T cells (1.4% ± 0.8; *n* = 6).

Remarkably, this function was not maintained in innate CD8(+) T cells from CML-CP patients, in which IL-12 + IL-18-induced intracellular IFN-γ expression was virtually undetectable [0.7% ± 0.1 (*n* = 6) vs. 0.4% ± 0.02 (*n* = 5) in their conventional/memory counterpart], as compared with HD (24.8% ± 1.2 (*n* = 6) vs. 1.4% ± 0.8 (*n* = 6) in their conventional/memory counterpart) (Figure 2A). In HD, innate CD8(+) T cells express more cytolytic activity than does their conventional/memory [KIR/NKG2A(−) Eomes(+)] CD8(+) counterpart, as attested by their more elevated perforin expression [71.4% ± 5.3 (*n* = 10) and 42.4% ± 9.1 (*n* = 6), respectively] (Figure 2B), and natural antibody-dependent cytotoxicity through CD16 as a lysis receptor [26.4% ± 6.2 (*n* = 6) and 11.4% ± 3.5 (*n* = 6), respectively] (Figure 2C).

As for intracellular IFN-γ expression, these two functions were once again dramatically reduced in innate CD8(+) T cells from CML-CP patients [44.0 ± 6.7% (*n* = 10) and 6.8% ± 4.1 (*n* = 5), respectively] all the way down to the levels found in the conventional/memory population [26% ± 13 (*n* = 6) and 5.1% ± 1.9 (*n* = 5), respectively] (Figures 2B,C).

Finally, both IFN-γ after IL-12 + IL-18 stimulation and cytolytic activity of classical NK cells were likewise decreased in CML-CP patients [7.0% ± 2.0 (*n* = 8) and 34.3% ± 11.5 (*n* = 5), respectively] as compared to HD [25.8% ± 3.9 (*n* = 15) and 65.9% ± 8.1 (*n* = 6), respectively] (Figure S2 in Supplementary Material). These findings, together with the similar frequencies of IFN-γ-expressing innate CD8(+) T cells, upon TCR engagement in HD (7.8% ± 6.4; *n* = 7) and CML-CP patients (9.1% ± 2.3; *n* = 7) (Figure S3 in Supplementary Material), would appear to imply that the functional deficiencies of innate CD8(+) T cells in CML-CP patients affect innate rather than adaptive immune responses.

### Partial Correction of Quantitative and Functional Deficiencies of Innate CD8(+) T Cells in Patients with CCyR

**Figure 3:**
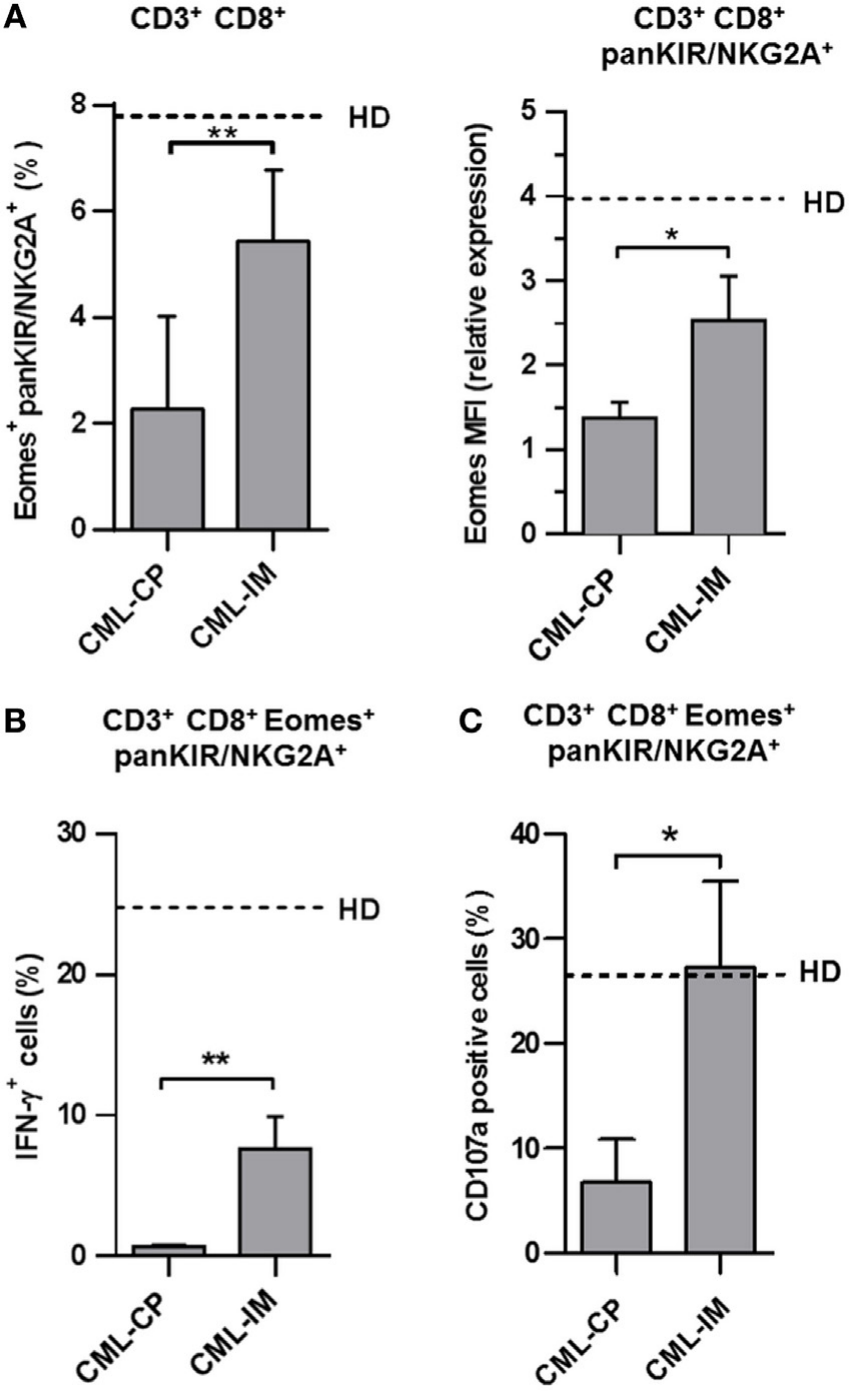
**The innate CD8 T cell subset is partially restored in patients having achieved complete cytogenetic remission with imatinib mesylate (IM) therapy**. **(A)** Partial restoration of the pool of innate CD8(+) T cells in chronic myeloid leukemia (CML)-IM. Frequency (mean ± SEM) of killer cell Ig-like receptor (KIR)/NKG2A(+) Eomes(+) cells among total CD8(+) CD3(+) cells (left panel) in CML-CP and CML-IM patients. Dotted lines represent mean frequency values in healthy donor (HD). Mean fluorescence intensity (MFI) of Eomes expression (mean ± SEM, right panel) in KIR/NKG2A(+) CD8(+) T cells from CML-CP and CML-IM patients were analyzed after gating on KIR/NKG2A(+) CD8(+) CD3(+) cells. MFI of Eomes expression was normalized on MFI of Eomes from total KIR/NKG2A(−) CD3(−) cells. Dotted lines represent mean relative MFI Eomes values in HD. **(B)** Partial restoration of IL-12 + IL-18-induced IFN-γ expression by innate T CD8(+) cells from CML-IM patients. For details, see the caption of Figure 2A. Histograms represent frequencies (means ± SEM) of IFN-γ-expressing cells among KIR/NKG2A(+) Eomes(+) CD8(+) T cells after IL-12 + IL-18 stimulation in each group of patients and HD. Without stimulation, frequency of IFN-γ-expressing cells was lower than 0.1%. **(C)** Complete restoration of natural cytotoxic activity by innate T CD8(+) cells from CML-IM patients. CD107a degranulation/expression after CD16 triggering (for details, see Section “Materials and Methods”) in KIR/NKG2A(+) Eomes(+) CD8(+) CD3(+) cells was analyzed by flow cytometry among peripheral blood mononuclear cells preincubated for 48 h with IL-15. Data (means ± SEM) are expressed as frequencies of CD107a-expressing cells in KIR/NKG2A(+) Eomes(+) CD8(+) CD3(+) cells from CML-CP and CML-IM patients. Dotted lines represent mean frequency values in HD. **(A–C)** Statistical significance was determined by the two-tailed Mann–Whitney non-parametric test (comparisons between HD and CML-CP patient groups) or the two-tailed Wilcoxon non-parametric test [comparisons between KIR/NKG2A(+) Eomes(+) CD8(+) CD3(+) cells and KIR/NKG2A(−) Eomes(+) CD8(+) CD3(+) cells from HD or CML-CP patient groups]. *p < 0.05; **p < 0.01.

In patients having achieved complete cytogenetic remission following IM therapy, referred to as CML-IM patients, we observed a recovery of innate CD8(+) T cells (Figure 3A, left panel) in CML-IM patients (5.4% ± 0.5; *n* = 6 vs. 2.3% ± 0.6; *n* = 7 in CML-CP patients), back close to the proportions found in HD (7.8% ± 0.9; *n* = 16), while Eomes expression (Figure 3A, right panel) was increased in CML-IM patients (MFI: 2.5 ± 0.5; *n* = 6) as compared with CML-CP patients (1.4 ± 0.2; *n* = 7) and was partially restored relative to HD (MFI: 4.0 ± 0.5; *n* = 16). Accordingly, the proportion of cells expressing Eomes in the KIR/NKG2A(+) CD8 T cell subset was restored in CML-IM patients (42.7% ± 2.9; *n* = 6) as compared with CML-CP patients (26.3% ± 3.2; *n* = 6), reverting to the proportions found in HD (46.5% ± 4.6; *n* = 14) (Figure S1 in Supplementary Material).

Importantly, in these conditions, IFN-γ expression in response to IL-12 + IL-18 was partially restored in the innate CD8(+) T cell compartment from CML-IM patients (7.7% ± 2.3; *n* = 6 vs. 0.7% ± 0.1; *n* = 5 in CML-CP patients) relative to HD (24.8% ± 1.2; *n* = 6) (Figure 3B), while cytolytic activity, measured as frequencies of CD107a-expressing cells in KIR/NKG2A(+) Eomes(+) CD8(+) CD3(+) cells, returned to normal (27.3% ± 8.3; *n* = 6 vs. 6.8% ± 4.1; *n* = 5 in CML-CP patients), relative to HD (26.4% ± 6.2; *n* = 6) (Figure 3C).

### Positive Correlation between iNKT Cell PLZF Expression and Innate CD8(+) T Cell Eomes Expression

**Figure 4:**
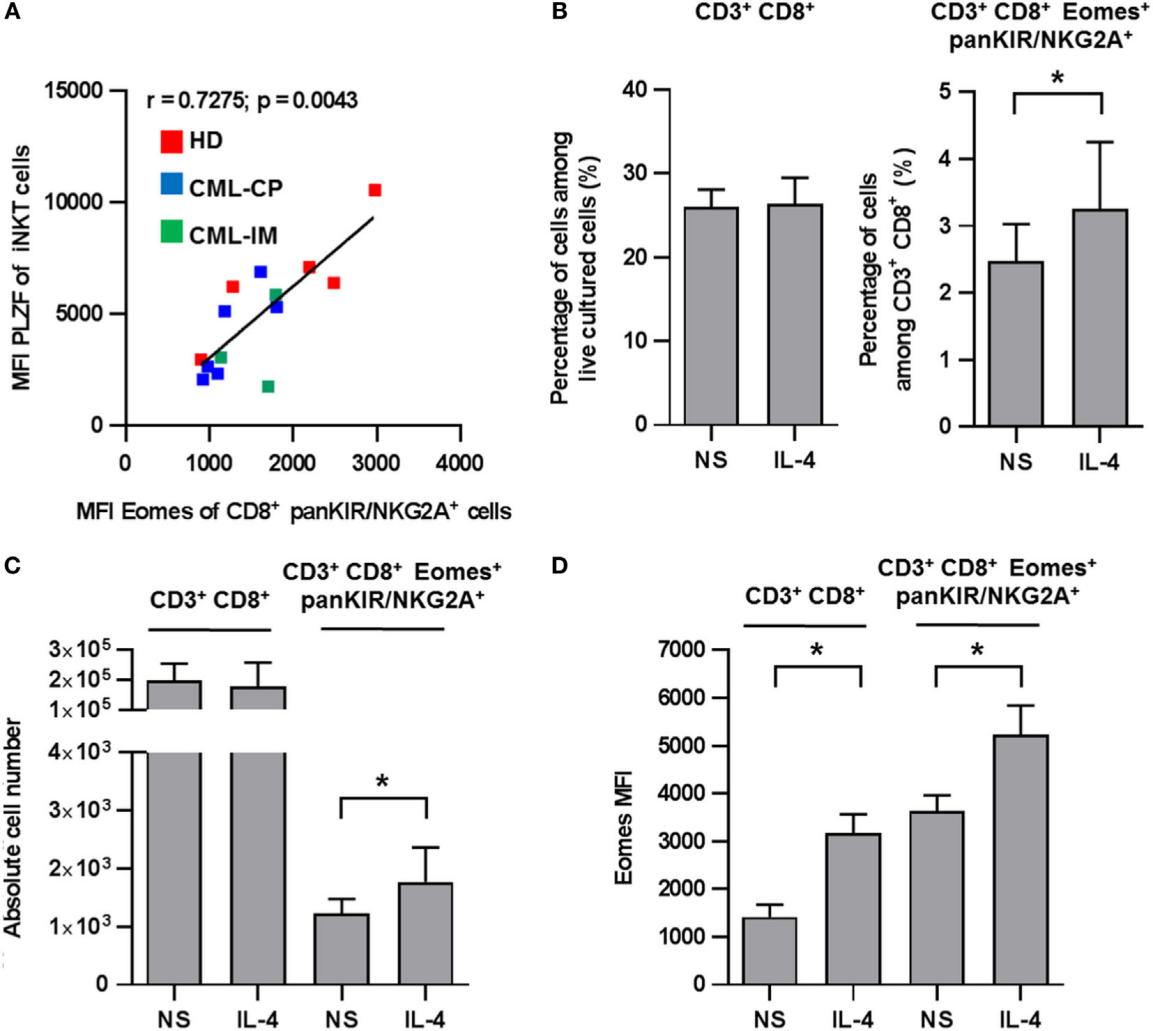
**(A)** Positive correlation between invariant natural killer T (iNKT) cell promyelocytic leukemia zinc finger (PLZF) expression and innate CD8 T cell Eomes expression. Eomes and PLZF expression were analyzed in innate CD8(+) T cells and iNKT cells, respectively, among peripheral blood mononuclear cells (PBMCs) by flow cytometry *ex vivo* after cellular permeabilization. Eomes expression and PLZF were analyzed after gating on killer cell Ig-like receptor (KIR)/NKG2A(+) CD8(+) CD3(+) cells and 6B11(+) CD3(+) cells, respectively. Mean fluorescence intensity (MFI) values are expressed relative to that of isotype control. Data from healthy donor (*n* = 5), chronic myeloid leukemia (CML)-CP (*n* = 6), and CML-imatinib mesylate (*n* = 3) were pooled. The MFI of PLZF-expressing iNKT [6B11(+) CD3(+)] cells correlate positively with MFI of Eomes expression on innate CD8(+) T [KIR/NKG2A(+) Eomes(+) CD8(+) CD3(+)] cells (correlation Spearman test; *n* = 14, *r* = 0.7275, *p* = 0.0043). **(B–D)** Innate CD8(+) T cells depend on IL-4 for homeostasis/expansion. PBMCs from five HDs were cultured for 7 days with IL-4 or medium alone (not stimulated, NS) and then analyzed by flow cytometry *ex vivo* after cellular permeabilization for innate CD8(+) T cells. Frequency **(B)**, absolute cell number **(C)**, and Eomes MFI **(D)** (mean ± SEM) of CD3(+) CD8(+) cells among total live PBMCs (left) and KIR/NKG2A(+) Eomes(+) CD8(+) CD3(+) cells among CD3(+) CD8(+) cells (right) are shown. Statistical significance was determined by the one-tailed Wilcoxon non-parametric test. *p < 0.05; **p < 0.01.

We have previously reported that IL-4 production by iNKT cells returned to normal in patients having achieved complete CML remission with TKI therapy (22). Given that, in mice, the differentiation of innate CD8(+) T cells depends mainly on PLZF-expressing iNKT cells *via* their IL-4 production (14, 15); we reasoned that the same phenomenon might be applied to humans. In accordance with this notion, we found a significant positive correlation between the levels of Eomes in KIR/NKG2A(+) CD8(+) T cells and of PLZF in iNKT cells including all the HD, CML-CP, and CML-IM samples available (Figure 4A). Moreover, we found that after 7 days of culture in the presence of IL-4, recovery of CD8(+) T cells was slightly, but significantly, increased both in terms of frequency and numbers as compared to the total CD3(+) CD8(+) cells (Figures 4B,C). We also confirmed in humans that IL-4 strongly enhances Eomes expression both in total CD3(+) CD8(+) cells and in innate CD8(+) T cells (Figure 4D). Taken together, these findings support the possible involvement of iNKT cells through their IL-4 production in the generation/maintaining of innate CD8(+) T cells in CML patients.

## Discussion

Although the concept of innate memory CD8(+) T cells is now well established in mice, whether an equivalent T-cell population exists in humans remains under debate. We recently reported that CD8(+) T cells co-expressing Eomes and KIR/NKG2A may represent a new, functionally distinct innate T cell subset in humans. Here, by extending our study to CML, we report that this disease is associated with an acquired and reversible defect of innate CD8(+) T cells. To the best of our knowledge, these findings are the first providing insights into the potential role of innate CD8(+) T cells in a physiopathological context in humans.

The finding that CML, at diagnosis is closely associated with a profound quantitative and functional deficiency in the innate CD8(+), T cell pool is in line with our hypothesis that this new subset may be involved during tumorigenesis in humans. This assumption was earlier based on our demonstration of terminally differentiated effector features of innate CD8(+) T cells in humans, such as rapid production of IFN-γ and induction of cytolytic function upon stimulation *in vitro*. In the present study, our revelation of the profound impact of CML on these potential antitumoral functions of innate CD8(+) T cells, together with those of NK (see Figure S2 in Supplementary Material) and iNKT cells (22, 27), support a role in cancer immune surveillance of innate CD8(+) T cells analogous to the two other innate cell pools. Even though these findings corroborate the concept that innate CD8(+) T cells, like iNKT cells, may act against tumors in a TCR-independent and NK-like manner, the possibility that these cells recognize leukemia cells cannot be excluded and deserves further investigation.

Remarkably, deficiencies of innate CD8(+) T cells found at diagnosis in CML patients were significantly reversed upon remission following TKI therapy. These data are consistent with the hypothesis that reconstitution of the pool of innate CD8(+) T cells and its partial functional restoration together with those of NK cells and iNKT cells effectively contribute to disease control of CML during TKI therapy. To definitively confirm this assumption, it will be important to further evaluate whether the emergence of innate CD8(+) T cells in terms of both number and functional potential is closely related to long-term remission after treatment discontinuation.

To date, in mice, even though the effector functions of innate/memory CD8(+) T cells have been extensively tested *in vitro*, their actual role during immune responses remains poorly documented. Testing the *in vivo* response following infection with the bacteria *Listeria monocytogenes*, innate/memory CD8(+) T cells were found to have a protective function (5, 6). As far as immune responses against tumors are concerned, only in lymphodepleted mice, does there exist evidence that these cells can enhance response to tumors (30–33). Given our findings, it remains to be directly determined, using experimental tumor models in mice, whether or not innate CD8(+) T cells exert protective functions.

The precise mechanisms for the generation and/or maintenance of innate CD8(+) T cells in humans remain to be determined. In the mouse, a key role in Eomes upregulation has been attributed to PLZF-expressing T cells. Similarly, our data in peripheral blood show a close correlation between the levels of Eomes in innate CD8(+) T cells and of PLZF in iNKT cells. Moreover, expression of PLZF and Eomes in iNKT cells and innate CD8(+) T cells, respectively, were both found to be reduced in patients at CML diagnosis, and reversed after disease remission, as were innate CD8(+) T cells in terms of both number and functions. Taken together, these findings are consistent with the existence of a PLZF-dependent mechanism in humans, similar to that reported in mice. It remains to determine whether, as in mice, PLZF-expressing T cells, especially iNKT cells, promote the development of blood peripheral innate T CD8(+) T cells by providing IL-4. In accordance with this view, we recently demonstrated a functional deficiency of IL-4 production by iNKT cells in CML patients at diagnosis and its partial restoration in patients having achieved remission after TKI therapy (22).

Further investigations are required to elucidate the exact mechanisms accounting for the deficiency of innate CD8(+) T cells at diagnosis and the beneficial effect of TKI therapy on this pool of cells. Nonetheless, from our data, it is tempting to speculate that the dysfunctions of innate CD8(+) T cells in CML patients at diagnosis that are remedied by TKI therapy originate from antigen APC-dependent dysfunctions, which in turn might contribute to iNKT cell deficiencies. This hypothesis arises from our recent findings showing that BCR-ABL from CML myeloid DCs mediates iNKT-cell immune subversion by downregulating cell-surface CD1d expression (27). Another non-exclusive mechanism may involve IL-15, which is required for maintenance not only of conventional memory CD8(+) T cells (34, 35) but also innate/memory CD8(+) T cells (32, 36). Indeed, CD8α(+) DC trans-presentation of IL-15 contributes to development of innate-like CD8(+) T cells in the periphery (37). For these reasons, the possible existence of a deficit in IL-15 expression and/or in IL-15 trans-presentation by mDCs from CML-CP patients is deserving of special attention.

All in all, by revealing an iNKT/innate CD8(+) T-cell axis with expected physiopathological relevance in CML, our findings should enhance understanding of the T cell components restored in CML treatment that contribute to disease control. From a more general standpoint, our study underscores the potential role of innate CD8(+) T cells against tumors in conjunction with iNKT cells and may contribute significantly to our understanding of the role of these innate T cell subsets in the development of protective immunity in humans.

## Author Contributions

FJ, EC, and AB designed the experiments, performed the experiments, analyzed and interpreted the data, and wrote the manuscript. DD, AL, and SB designed the experiments, performed the experiments, and analyzed and interpreted the data. AR, NP, and LL provided assistance with cell cultures. CG contributed to PBMC preparation from patients and healthy controls. FG and LR provided clinical samples and contributed to the interpretation of data. AH and J-MG together were responsible for the overall study design, supervised the project, and took primary responsibility for writing the manuscript.

## Conflict of Interest Statement

The authors declare that the research was conducted in the absence of any commercial or financial relationships that could be construed as a potential conflict of interest.
