# Supplementary figures and images for: The Hypothesis of the Human iNKT/Innate CD8(+) T-Cell Axis Applied to Cancer: Evidence for a Deficiency in Chronic Myeloid Leukemia
Source: Front Immunol. 2017 Jan 16;7:688. doi: 10.3389/fimmu.2016.00688 (PMC5237805; doi:10.3389/fimmu.2016.00688)

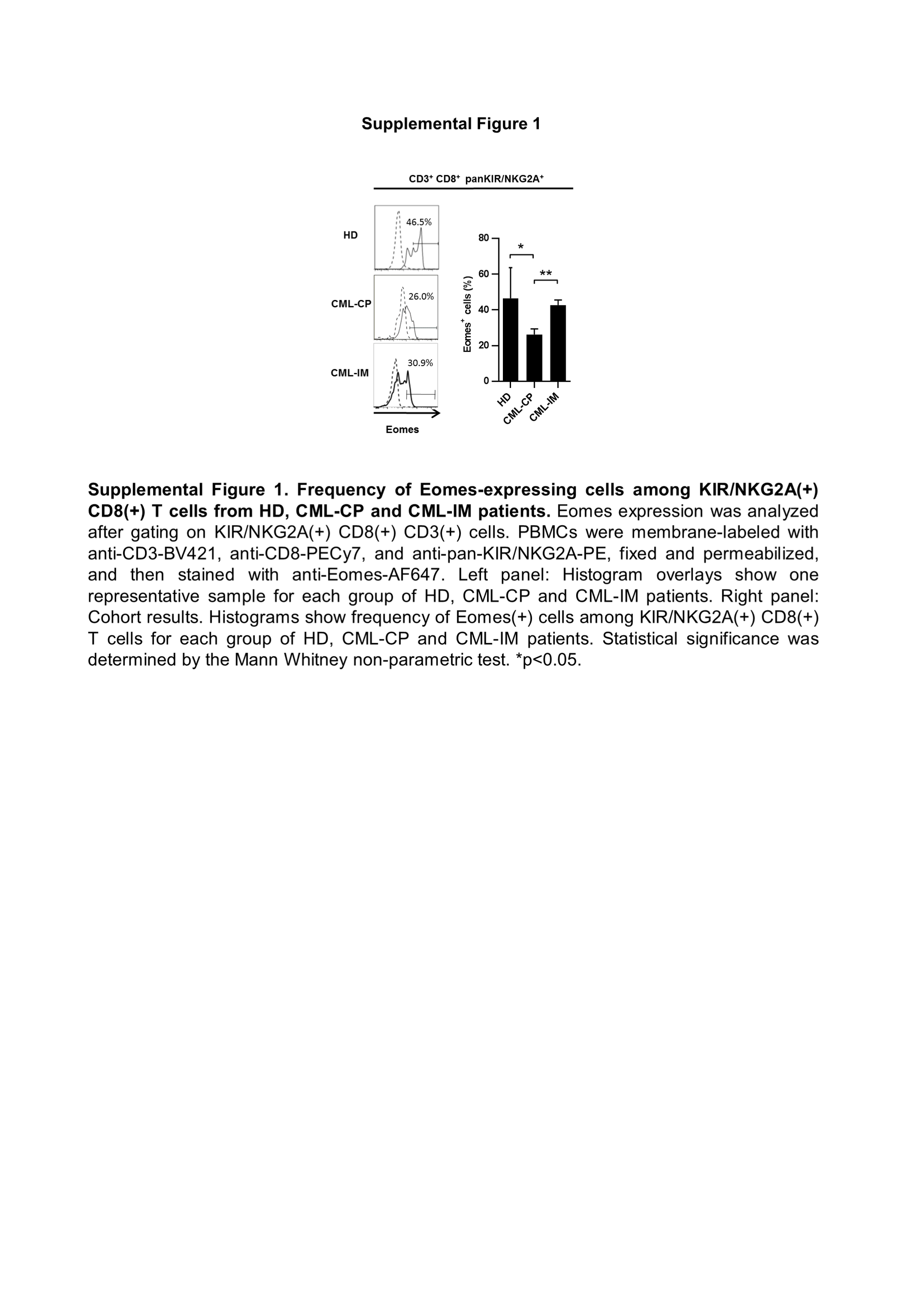

Supplement: Supplementary file 1 [file image_1.tif]

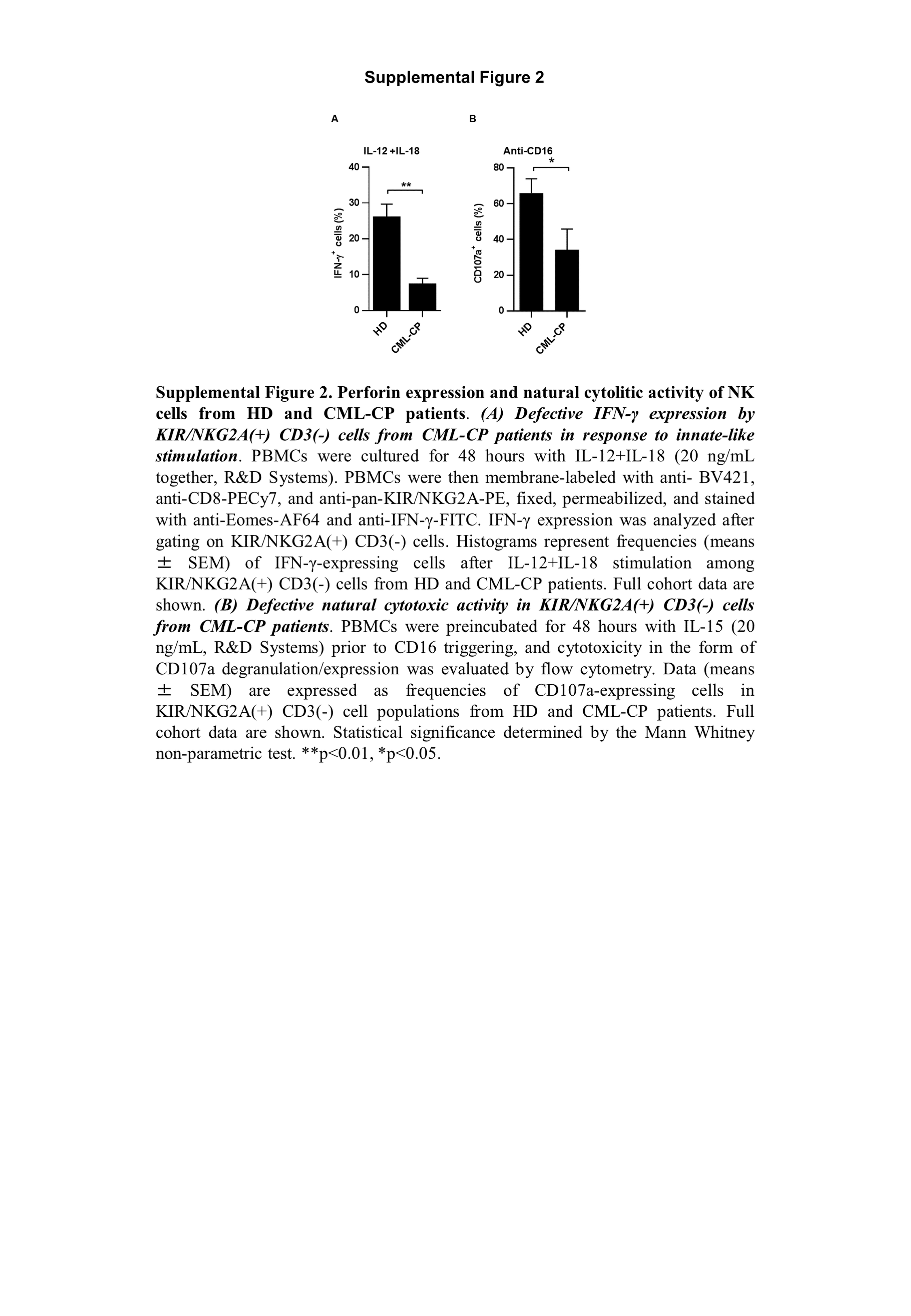

Supplement: Supplementary file 2 [file image_2.tif]

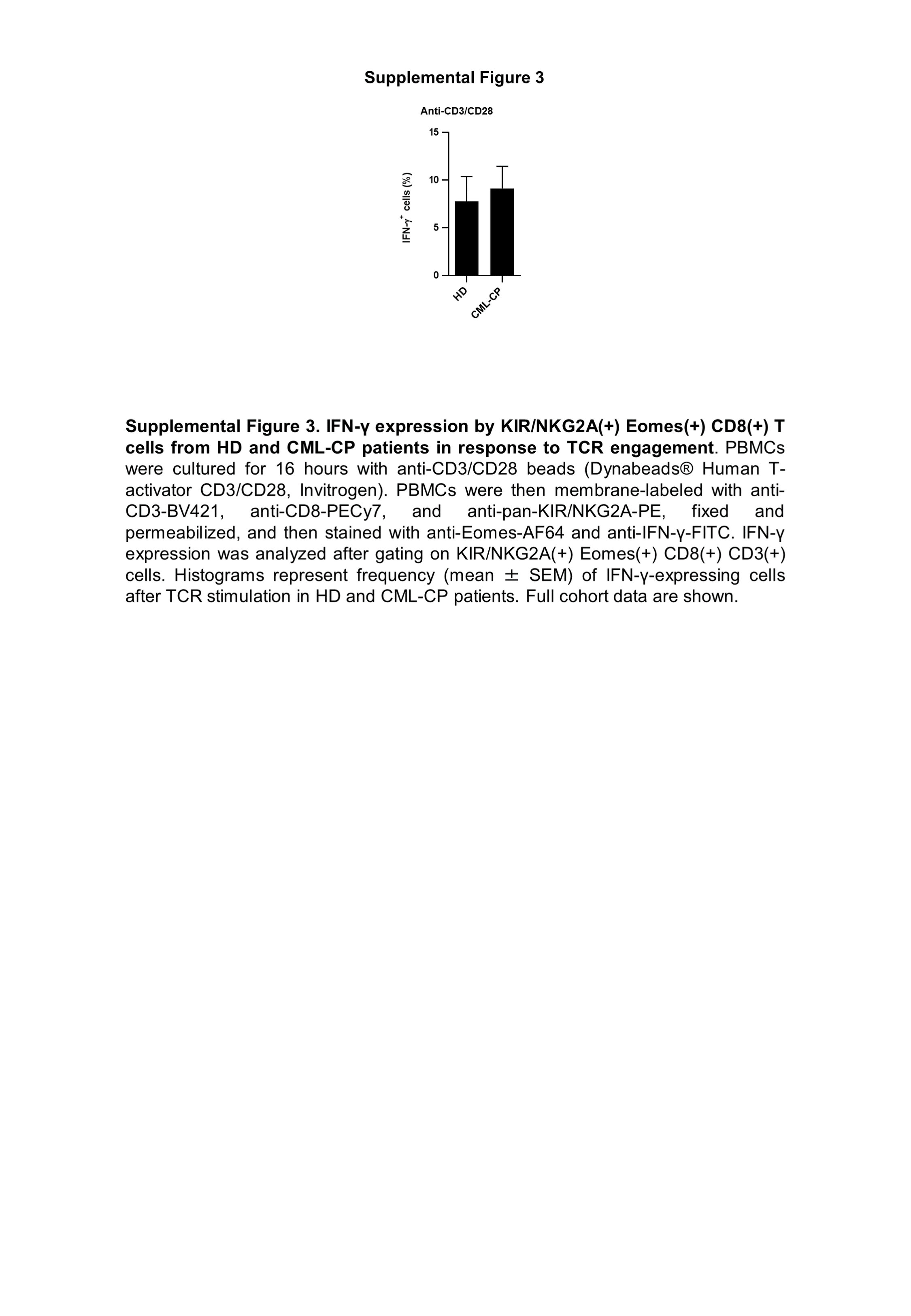

Supplement: Supplementary file 3 [file image_3.tif]
